# CD9, a potential leukemia stem cell marker, regulates drug resistance and leukemia development in acute myeloid leukemia

**DOI:** 10.1186/s13287-021-02155-6

**Published:** 2021-01-25

**Authors:** Yongliang Liu, Guiqin Wang, Jiasi Zhang, Xue Chen, Huailong Xu, Gang Heng, Jun Chen, Yongchun Zhao, Jiatao Li, Yuanli Ni, Yingzi Zhang, Juanjuan Shan, Cheng Qian

**Affiliations:** 1 Center of Biological Therapy, Southwest Hospital, Army Medical University, Chongqing, China; 2 Center for Precision Medicine of Cancer, Chongqing Key Laboratory of Translational Research for Cancer Metastasis and Individualized Treatment, Chongqing University Cancer Hospital, Chongqing, China; 3 Department of Hematology, Southwest Hospital, Army Medical University, Chongqing, China; 4 Chongqing Institute of Precision Medicine and Biotechnology Co., Ltd., Chongqing, China

**Keywords:** Acute myeloid leukemia (AML), Leukemia stem cells (LSCs), CD9, Alpha-2-macroglobulin (A2M), Biomarker

## Abstract

**Background:**

Leukemia stem cells (LSCs) are responsible for the initiation, progression, and relapse of acute myeloid leukemia (AML). Therefore, a therapeutic strategy targeting LSCs is a potential approach to eradicate AML. In this study, we aimed to identify LSC-specific surface markers and uncover the underlying mechanism of AML LSCs.

**Methods:**

Microarray gene expression data were used to investigate candidate AML-LSC-specific markers. CD9 expression in AML cell lines, patients with AML, and normal donors was evaluated by flow cytometry (FC). The biological characteristics of CD9-positive (CD9^+^) cells were analyzed by in vitro proliferation, chemotherapeutic drug resistance, migration, and in vivo xenotransplantation assays. The molecular mechanism involved in CD9^+^ cell function was investigated by gene expression profiling. The effects of alpha-2-macroglobulin (A2M) on CD9^+^ cells were analyzed with regard to proliferation, drug resistance, and migration.

**Results:**

CD9, a cell surface protein, was specifically expressed on AML LSCs but barely detected on normal hematopoietic stem cells (HSCs). CD9^+^ cells exhibit more resistance to chemotherapy drugs and higher migration potential than do CD9-negative (CD9^−^) cells. More importantly, CD9^+^ cells possess the ability to reconstitute human AML in immunocompromised mice and promote leukemia growth, suggesting that CD9^+^ cells define the LSC population. Furthermore, we identified that A2M plays a crucial role in maintaining CD9^+^ LSC stemness. Knockdown of A2M impairs drug resistance and migration of CD9^+^ cells.

**Conclusion:**

Our findings suggest that CD9 is a new biomarker of AML LSCs and is a promising therapeutic target.

**Supplementary Information:**

The online version contains supplementary material available at 10.1186/s13287-021-02155-6.

## Background

Acute myeloid leukemia (AML) is the most common acute leukemia in adults, accounting for approximately 80% of cases in this group, and is characterized by infiltration of the bone marrow, blood, and other tissues by proliferative, clonal, abnormally differentiated, and occasionally poorly differentiated cells of the hematopoietic system [1, 2]. AML is caused by disorders of the hematopoietic system and is often treated with chemotherapeutic and/or hematopoietic stem cell transplantation [3]. However, 43% of young adult patients eventually relapse after achieving complete remission [4]. Residual leukemia stem cells (LSCs), a rare cell type, are the major cause of recurrence of AML and possess chemoresistance and the ability to self-renew and differentiate, thus reconstituting AML. Therefore, the LSC theory has inspired the design of innovative treatment strategies for AML aimed at targeting LSCs hidden in leukemia.

To identify potential therapeutic targets, many groups have reported cell surface proteins that are preferentially expressed on AML LSCs, including CD47 [5], CD44 [6], CD96 [7], CD123 [8, 9], CD99 [10], and TIM-3 [11, 12]. During the past few years, some strategies for targeting LSC antibodies or immune cells have already been tested in patients but still face problems relating to toxicity and LSC resistance. Therefore, more specific LSC markers still need to be explored.

Here, we identified CD9 as a potential AML LSC-specific molecule by analyzing three microarray datasets of AML LSCs and conducting minimal residual disease (MRD) expression profiling. As a member of the tetraspanin family, CD9 is the third most abundant protein on the surface of platelets and is required for the release of microparticles from coated-platelets [13, 14]. Furthermore, it was reported that CD9 plays an important role in cell adhesion, movement, differentiation, proliferation, apoptosis, and chemotherapy resistance [15–19]. CD9 has been described as a cancer stem cell marker in several types of cancers, including pancreatic cancer, glioblastoma, and B-acute lymphoblastic leukemia, and is related to the prognosis of AML [15, 20–22]. However, the biological characteristics and regulatory mechanism of CD9^+^ AML LSCs remain to be elucidated.

In this study, we found high expression of CD9 in AML patient LSCs and extremely low expression in normal hematopoietic stem cells (HSCs). CD9^+^ cells exhibited stem cell characteristics, including drug resistance and migration ability, and could establish human AML in immunocompromised mice. Mechanistically, we identified by transcription profiling analysis that A2M plays a crucial role in CD9^+^ LSC maintenance. Downregulation of A2M impairs the drug resistance and migration ability of CD9^+^ cells. In summary, our data suggest that CD9 is a potential new target for AML therapy and that A2M controls the stemness characteristics of CD9^+^ AML LSCs.

## Materials and methods

### Data sources

Three AML LSC sequencing chips and one AML minimal disease residue (MDR) sequencing chip were sourced from a publicly available database (Microarray 1, (GSE24006) https://www.ebi.ac.uk/arrayexpress/experiments/E-GEOD-24006/?query=GSE24006; Microarray 2, (GSE24395) https://www.ebi.ac.uk/arrayexpress/experiments/E-GEOD-24395/?query=GSE24395, and Microarray 3, https://www.ncbi.nlm.nih.gov/pmc/articles/PMC3005290/; MRD, https://insight.jci.org/articles/view/98561/sd/3.

### Cell lines and leukemic cells from patients

THP-1 and KG-1α cells were obtained from American Type Culture Collection (ATCC); U937 cells were obtained from the Chinese Academy of Sciences, Shanghai, China; MV-4-11 cells were obtained from the Query Network for Microbial Species of China; MOLM-13 cells were obtained from COBIOER Biosciences, Nanjing, China; and HL-60 cells were obtained from JOINN Laboratories, Suzhou, China.

Primary AML cells were obtained from the bone marrow of patients with AML who signed informed consent forms according to protocols approved by the Institutional Review Board of the Southwest Hospital, Army Medical University. Patient information is presented in supplementary Table S1. Normal bone marrow samples were obtained from volunteers.

Primary AML cells were cultured in RPMI 1640 (Gibco) medium supplemented with 20% FBS, 100 U/ml penicillin, 0.1 mg/ml streptomycin, and 10 ng/ml each of the human cytokines IL-3, Flt-3 ligand, TPO and SCF (PeproTech).

### Antibodies, cell staining, and sorting

All antibodies for FC were purchased from BioLegend. For analyses of CD9 expression in AML cell lines, cells were stained with PE anti-human CD9 antibody (HI9a). For analyses in primary AML cells, cells were stained with FITC anti-human CD3/CD19 antibody (HIT3a, 4G7), PerCP anti-human CD45 antibody (2D1), APC/Cyanine7 anti-human CD34 antibody (561), APC anti-human CD38 antibody (HB-7), and PE anti-human CD9 antibody (HI9a). Briefly, cells were harvested and suspended in 50 μl of staining/washing buffer (PBS including 1% FBS), stained with the indicated antibodies, and incubated for 30 min at 4 °C. Cells were washed with staining/washing buffer and suspended in buffer for FC or cell sorting.

### Migration assay

A Falcon® Permeable Support with an 8.0-μm Transparent PET Membrane (Corning) was used for the migration assay. A total of 2 × 10^5^ CD9^+^ or CD9^−^ cells sorted from cell lines or primary AML cells were suspended in 200 μl of RPMI 1640 medium (without FBS) and seeded in the upper chambers of supports in 24-well plates. Then, 900 μl of medium with 20% FBS was added to the bottom chamber of each well. After a 6-h incubation, migrated cells were counted by trypan blue exclusion staining at the indicated time points [23].

### Drug resistance assay

The drug resistance of AML cells was assessed by the MTS assay. AML cells (1 × 10^5^) were seeded in 96-well plates (NEST) and cultured with different concentrations of cytarabine (10 and 100 μg/ml) in 100 μl of medium [24]. After 24 h of incubation, 10 μl of MTS (Promega) was added to each well. After another 2 h of incubation, the absorbance of each well in the plate was measured at a wavelength of 490 nm with a microplate reader (Thermo Fisher Scientific).

### Cell proliferation assay

AML cells were seeded in 96-well plates at 5 × 10^3^ cells/well. At the indicated time, 10 μl of MTS (Promega) was added to cells, and then, the absorbance was detected at 490 nm after 2 h with a microplate reader (Thermo Fisher Scientific). Medium without cells was used as a negative control [25].

### Transplantation of AML cells into immunodeficient mice

All animal experiments were performed in accordance with the protocol approved by the Institutional Animal Care and Use Committee of Southwest Hospital, Army Medical University. Female NOG mice (Vital River Laboratories) aged 6 to 8 weeks were used for xenogeneic transplantation assays. THP-1 cells were infected with lentivirus coexpressing luciferase and GFP according to a previous method [26]. A total of 1X10^6^ sorted CD9^+^GFP^+^ and CD9^−^GFP^+^ THP-1 cells were transplanted into NOG mice (6 mice/group) via tail vein injection. The progression of leukemia was monitored by bioluminescence imaging with In Vivo Imaging System (IVIS) Spectrum (Perkin Elmer, USA) and Living Image Software for IVIS (Perkin Elmer).

### Microarray analysis

Three pairs of sorted CD9^+^ and CD9^−^ primary AML cells (patient 6, patient 7, and patient 8) were evaluated with the BGISEQ-500 platform to determine gene expression patterns. In brief, total RNA was extracted from sorted CD9^+^ and CD9^−^ primary AML cells with TRIzol (Invitrogen) according to the manufacturer’s instructions. Then, all samples were submitted to the BGISEQ-500 platform for RNA sequencing.

### Real-time PCR

Total RNA was extracted from CD9^+^ and CD9^−^ THP-1 cells using RNAiso Plus (Takara), and then, single-strand cDNA was synthesized with the PrimeScript RT Reagent Kit (Takara) according to the manufacturer’s instructions. Quantitative PCR analysis was performed using SYBR premix Ex Taq (Takara). The sequences of the primers were all from Primer Bank (https://pga.mgh.harvard.edu/primerbank/).

### Western blotting

Cells were washed with ice-cold PBS and lysed with RIPA buffer supplemented with protease and phosphatase inhibitor cocktail (Roche). The primary antibody for GAPDH (2118) was purchased from Cell Signaling Technology, for A2M (ab58703) was purchased from Abcam, and for EGR1 (100899-T32) was purchased from Sino Biological.

### shRNA-mediated A2M knockdown

THP-1 and HL-60 cells were infected with lentiviruses expressing shRNAs (sh-A2M 1, 5′-CCTAACATCTATGTACTGG-3′; sh-A2M 2, 5′-ATAGTGAAAGTCTATGATT-3′; and sh-Control, 5′-TTCTCCGAACGTGTCACGT-3′). Briefly, concentrated virus (MOI of 50) was directly added to cells in the presence of 8 μg/ml polybrene, and spinoculation was conducted at 32 °C and 800×*g* for 60 min.

### A2M network with CD9 based on the GeneMANIA database

Datasets, including physical interactions, pathways, and genetic interactions, were collected from the GeneMANIA public database. The dataset relevant to the A2M and CD9 networks was produced from the GeneMANIA database (http://www.genemania.org).

### Chromatin immunoprecipitation

Chromatin immunoprecipitation (ChIP) assays were performed according to the manufacturer’s instructions (Cell Signaling Technology, 9005S). Anti-EGR1 antibody was purchased from Cell Signaling Technology (4154S). The PCR primers for the CD9 promoter are listed in supplementary Table S2.

### Statistical analysis

All experiments were repeated at least in triplicate. Collected data were analyzed with GraphPad Prism 8.0 software (GraphPad Software, Inc., San Diego, CA), and the estimated variation was considered for each group of data and indicated as the SEM or SD in each figure legend. Comparisons between two groups were carried out with unpaired Student’s *t* test (two tailed), and differences among more than two groups were determined by one-way ANOVA followed by the Newman-Keuls test. Differences with *p* < 0.05 were considered statistically significant.

## Results

### CD9 is highly expressed in the CD34^+^CD38^−^ cell population of AML patients and shows almost no expression in normal HSCs

**Fig. 1:**
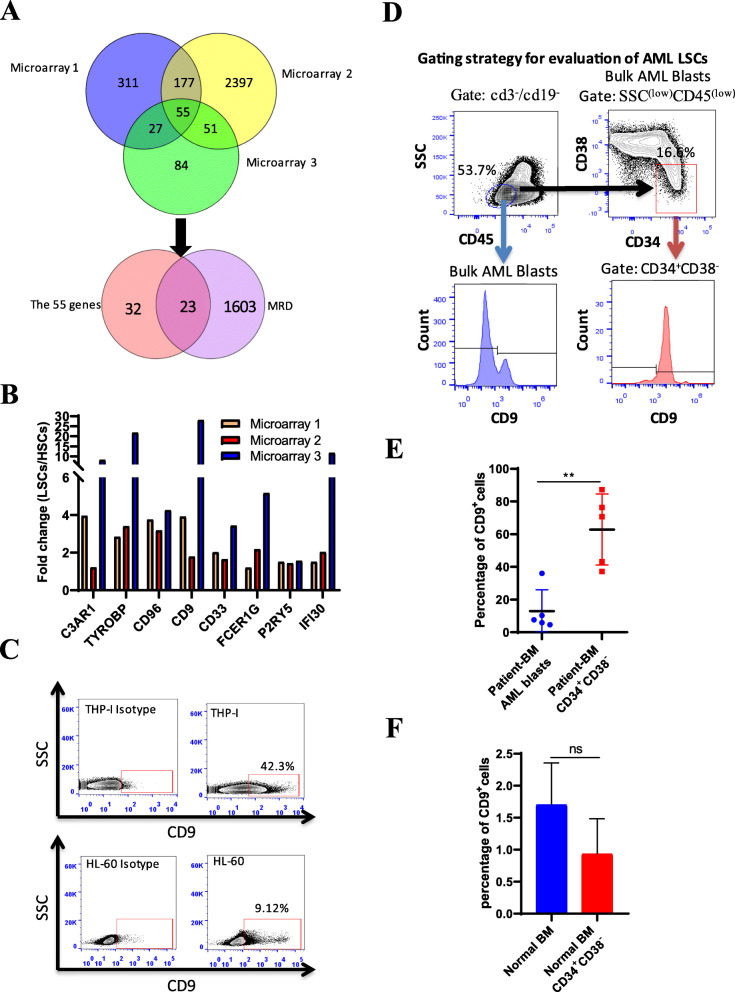
CD9 is enriched in AML-LSC population. **a** The Venn diagram compares the number of specifically expressed and shared differentially expressed genes among the three AML LSCs sequencing chips. Fifty-five common differentially expressed genes were analyzed in the MRD microarray by the Venn diagram. **b** The expression levels of 8 genes related to membrane proteins. **c** Flow cytometry was used to detect the expression of CD9 in the AML cell lines THP-1 and HL-60. **d** Gating strategy for comparing the expression of CD9 on AML blasts (CD3^−^CD19^−^CD45^low^SSC^low^) and LSC-enriched CD34^+^CD38^−^ populations (CD3^−^CD19^−^CD45^low^SSC^low^CD34^+^CD38^−^). **e** The expression of CD9 on AML blasts and LSC-enriched CD34^+^CD38^−^ populations from the bone marrow of patients with AML (*n* = 5). **f** CD9 expression in the normal bone marrow

To identify cell surface markers that are selectively expressed on AML LSCs, we surveyed three sets of AML LSC sequencing chip data [11, 27, 28]. Fifty-five commonly upregulated genes were detected by comparing the three datasets (Fig. 1a). MRD in AML patients refers to residual cancer cells after treatment, and it was reported to be a powerful and independent prognostic factor in treatment outcome [29–38]. We thus investigated whether the expression level of the fifty-five LSC upregulated genes was also upregulated in the MRD microarray. By analyzing the MRD microarray data, we found that among these fifty-five genes, twenty-three genes were significantly upregulated in the MRD microarray, including the cell surface proteins CD33, CD96, P2RY5, C3AR1, TYROBP, FCER1G, IFI30, and CD9 (Fig. 1a, b, supplementary Table S3). Interestingly, CD96 has already been reported as an LSC-specific marker in human AML [7], which strongly confirmed the reliability of our findings. Among these membrane proteins, CD9 was the most intensively upregulated gene. We first analyzed the level of CD9 expression in AML cell lines by flow cytometry (FC). The results showed that the percentages of CD9 expression ranged from 1.9 to 42.3% (Fig. 1c, S1). Then, we examined CD9 expression in the bone marrow of AML patients and normal donors. With the previously described gating strategy in FC, we first excluded CD3- and CD19-positive T cells and B cells, focused on the CD45^low^SSC^low^ population, analyzed CD34^+^CD38^−^ cells within CD45^low^SSC^low^, and then further investigated CD9 expression in CD3^−^CD19^−^CD45^low^SSC^low^CD34^+^CD38^−^ [10] (Fig. 1d). Our data revealed that the average percentage of CD9^+^ cells among AML blasts was 12.9% (5.84%–36%). It has been shown that AML LSCs mainly reside within the CD34^+^CD38^−^ fraction of leukemic cells. CD9^+^ cells accounted for 62.76% (37.2%–87.1%) of CD34^+^CD38^−^ cells (Fig. 1e), suggesting that CD9^+^ cells were enriched in AML LSCs. To determine whether CD9 expression could distinguish LSCs from normal HSCs, we examined CD9 expression in the bone marrow of normal donors. The results showed that CD9 expression was very low in normal bone marrow cells (1.7%, 1.1%–2.4%) and normal HSCs (0.9%, 0.3%–1.3%) (Fig. 1f). These data together demonstrate that the cell surface protein CD9 could be a promising marker for targeting AML LSCs.

### CD9^+^ cells exhibited LSCs characteristics

**Fig. 2:**
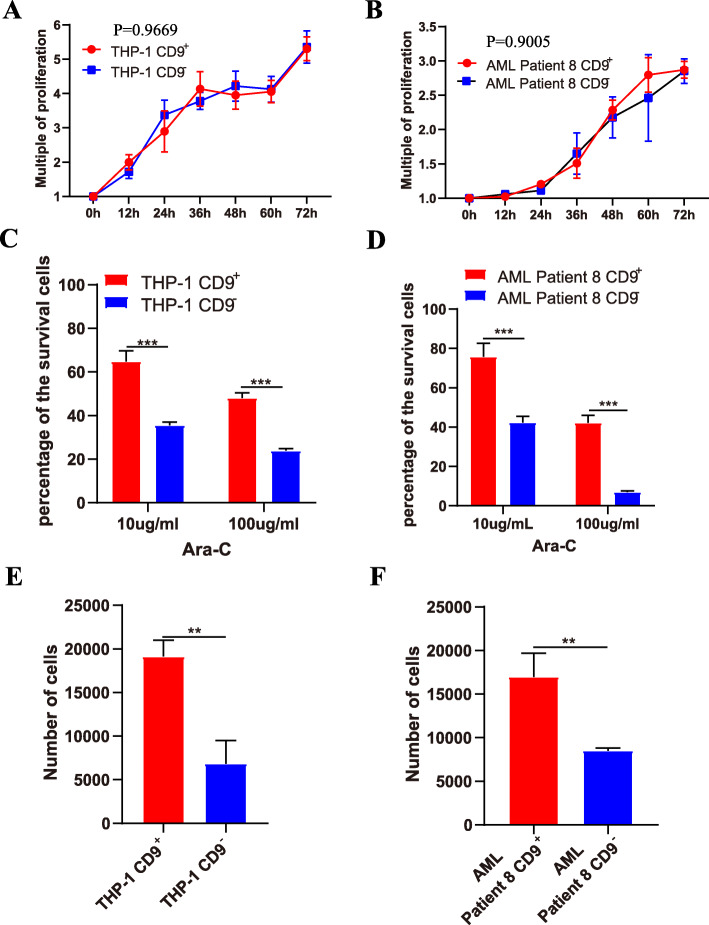
CD9^+^ cells exhibit stemness characteristics. **a**, **b** Proliferative ability of CD9^+^ cells and CD9^−^ cells. Error bars represent ±SD of triplicates. **c**, **d** Ara-C resistance of CD9^+^ cells and CD9^−^ cells. Error bars represent ±SD of triplicates. **e**, **f** Migration ability of CD9^+^ cells and CD9^−^ cells. Error bars represent ±SD of triplicates. * *P* < 0.05, **p < 0.01, ***p < 0.001

To investigate the biological function of CD9^+^ cells, we isolated CD9^+^ cells and CD9^−^ cells from THP-1 and AML patients by fluorescence-activated cell sorting. Cell proliferation assays showed that there was no significant difference in the proliferative capacity of CD9^+^ cells and CD9^−^ cells from either THP-1 cells or AML patients (Fig. 2a, b, *p* = 0.9669 and *p* = 0.9005, respectively), which is consistent with previous reports that stem cells do not exhibit superior proliferation capacity under normal conditions [39–41]. In addition, cell cycle analysis revealed that there were no differences between CD9^+^ and CD9^−^ cells in cell cycle progression (Fig. S2). To assess drug resistance, we treated the sorted cells with different doses of Ara-C (10 μg/ml and 100 μg/ml), a commonly used leukemia chemotherapy drug, and checked the cell survival rate after 24 h. The results showed that CD9^+^ cells were more resistant to Ara-C than were CD9^−^ cells (Fig. 2c, d). Furthermore, the Transwell migration assay showed that CD9^+^ cells exhibited higher migration potential than did CD9^−^ cells (Fig. 2e, f).

**Fig. 3:**
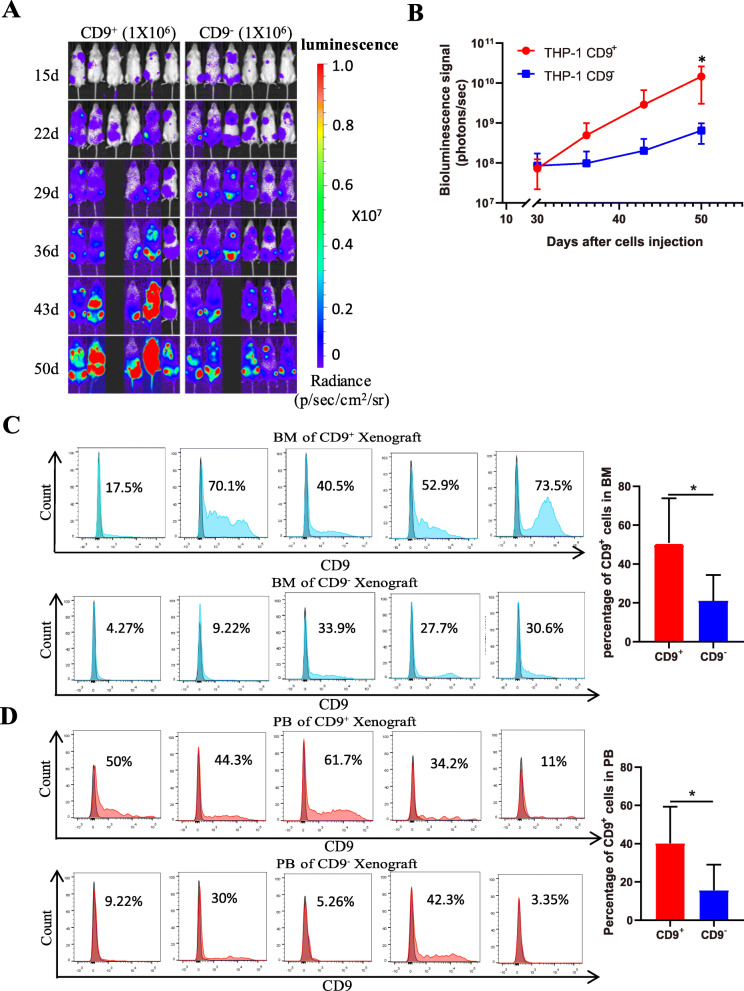
CD9^+^ cells promote leukemia growth. **a** Bioluminescent images showed that the total flux of the leukemia derived from CD9^+^ THP-1 cells (left) and CD9^−^ THP-1 cells (right) after tail vein injected in NOG mice. Signal intensity is represented as p/s/cm^2^/sr. **b** Quantification showed that the total flux of the leukemia derived from CD9^+^ THP-1 cells and CD9^−^ THP-1 cells after tail vein injected in NOG mice (*n* = 6). **c** The proportion of CD9^+^ cells in mice bone marrow. Error bars show the ±SD. **d** The proportion of CD9^+^ cells in mice peripheral blood. Error bars show the ±SD

To study the function of CD9^+^ AML cells in vivo, THP-1 cells were stably infected with lentivirus coexpressing luciferase and GFP to facilitate subsequent observation of leukemia growth in vivo. A total of 1 × 10^6^ CD9^+^GFP^+^ cells or CD9^−^GFP^+^ cells from the THP-1 cell population were injected into NOG mice via tail vein. The results showed that CD9^+^ cells exhibited superior proliferation capacity compared to that of CD9^−^ cells in vivo (Fig. 3a, b). The proportion of CD9^+^ cells in the bone marrow of mice was tested by FC, and the results showed that the bone marrow from mice injected with CD9^+^ cells contained a large number of infiltrating CD9^+^ cells (50.9%, 17.5%–73.5%). Unexpectedly, varying degrees of CD9^+^ cells were also found in the bone marrow of mice injected with CD9^−^ cells (21.02%, 4.27–33.9%) (Fig. 3c), which may explain why CD9^−^ mice can also develop leukemia. In addition, the existence of the same phenomenon in the peripheral blood of mice was observed (Fig. 3d). Previous studies have shown that cancer stem cells and differentiated tumor cells can be transformed into each other in the tumor microenvironment [42, 43]. Furthermore, survival analysis showed that mice transplanted with THP-1 CD9^+^ cells survived for a shorter period of time than did mice transplanted with THP-1 CD9^−^ cells (Fig. S3). In conclusion, CD9^+^ cells display LSC characteristics, exhibit drug resistance, present an increased capacity of migration, and promote leukemia progression.

### A2M is expressed in CD9^+^ cells at high levels

**Fig. 4:**
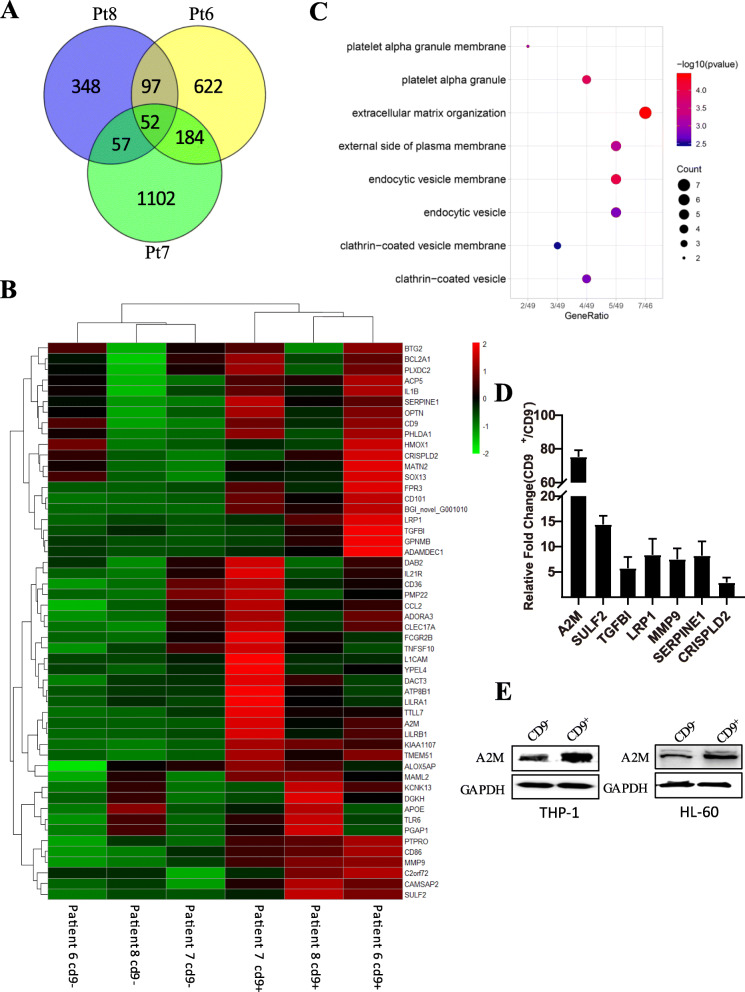
High expression of A2M in CD9^+^ AML LSCs. **a** Venn diagram analysis of RNA-seq results from AML-LSCs (CD9^+^) and non-LSCs (CD9^−^) of three AML patients. **b** Heatmap showing 52 common differentially expressed genes in the three AML patients. **c** Gene ontology analysis of genes in the heatmap. **d** The expression levels of 7 genes associated with extracellular matrix organization were detected by RT-PCR. **e** The expression levels of A2M in CD9^+^ cells than CD9^−^ cells are detected by Western blotting

To investigate the molecular mechanisms involved in CD9^+^ LSC maintenance, we established global gene expression profiles in CD9^+^ cells and CD9^−^ cells from three AML patients by cDNA microarray. A Venn diagram was used to analyze the differentially expressed genes that were upregulated in these 3 gene sets, and 52 differentially expressed genes that were commonly upregulated in the CD9^+^ population were initially detected (Fig. 4a, b).

We further performed gene ontology analysis of the 52 genes, among which the top cluster comprised genes involved in extracellular matrix organization, including A2M, SULF2, TGFBI, LRP1, MMP9, SERPINE1, and CRISPLD2 (Fig. 4c). We then confirmed the expression levels of these extracellular matrix-associated genes in THP-1 cells by real-time PCR (Fig. 4d). We focused on A2M not only because its expression level in CD9^+^ cells was high but also because activation of A2M signaling was reported to promote the proliferation and survival of cancer cells [44]. We confirmed the elevated protein expression of A2M in THP-1 and HL-60 CD9^+^ cells by Western blotting (Fig. 4e).

### A2M regulates CD9^+^ LSC maintenance

**Fig. 5:**
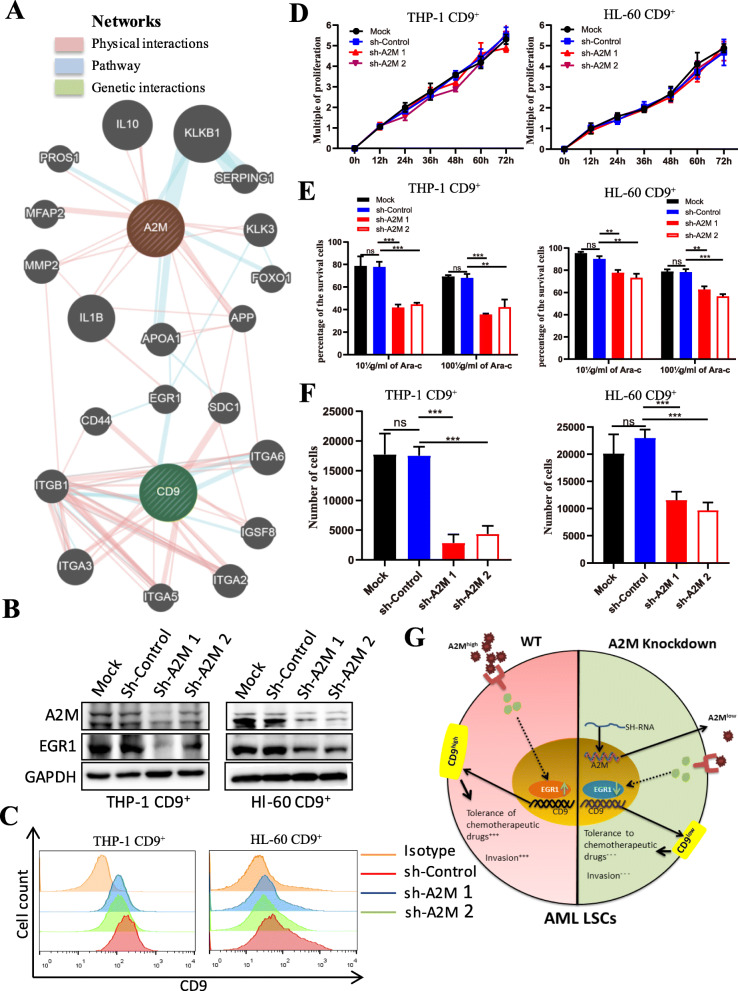
A2M regulates the stem cell characteristics of CD9^+^ cells. **a** The interactions of CD9 and A2M in the network were analyzed by GeneMANIA using the parameters limited to physical interactions, genetic interactions, and pathways to score nodes and source organism *Homo sapiens* as additional parameters. **b** Protein expression of A2M and EGR1 in CD9^+^ cells after A2M knockdown. **c** CD9 expression after A2M knockdown. (THP-1: sh-Control = 78.66%, sh-A2M 1 = 53.2%, sh-A2M 2 = 51.89%; HL-60: sh-Control = 58.62, sh-A2M 1 = 34.3%, sh-A2M 2 = 31.6%). **d** The proliferation ability of CD9^+^ cells after A2M knockdown. Error bars represent ±SD of triplicates. **e**, **f** The drug-resistant ability and migration ability of CD9^+^ cells after A2M knockdown. Error bars represent ±SD of triplicates. **g** Schematic summary of the role of A2M in regulating the stemness of CD9^+^ AML-LSCs. *p < 0.05, **p < 0.01, ***p < 0.001

To further investigate the connection between CD9 and A2M, the GeneMANIA webserver was applied to predict their interactions in the network with the parameters limited to physical interactions, genetic interactions, and pathways to score nodes; the source organism *Homo sapiens* was set as an additional parameter (Fig. 5a). From the GeneMANIA network, we found that A2M networked with CD9. To test whether A2M regulates CD9 expression, the expression of A2M in CD9^+^ cells was knocked down by short hairpin RNA (shRNA). The results showed that A2M knockdown significantly reduced the expression levels of EGR1 and CD9 (Fig. 5b, c). EGR1, as an important transcription factor, is a node in the A2M and CD9 network (Fig. 5a). The results revealed that A2M possibly regulates CD9 expression by regulating its downstream protein EGR1, and this conclusion was confirmed by ChIP (Fig. S4). Functionally, although knockdown of A2M had no effect on the proliferation of CD9^+^ cells (Fig. 5d), compared with the control groups, it significantly increased the sensitivity of CD9^+^ cells to Ara-C treatment and attenuated CD9^+^ cell migration (Fig. 5e, f). Therefore, we concluded that A2M is an upstream gene that regulates CD9 gene expression through EGR1 and controls AML LSC characteristics (Fig. 5g).

## Discussion

Cancer stem cells (CSCs) drive tumor initiation, progression, and metastasis. AML is a clonal malignant disorder derived from a small number of LSCs. LSCs could be the ultimate cellular target to cure human AML. Scientists are dedicated to searching specific LSC markers that can effectively distinguish between LSCs and normal HSCs. Many molecules were reported to be differentially expressed on AML LSCs, such as CD47, CD44, CD96, TIM3, CD99, and CD123 [5–7, 9–11]; however, some of these markers are not specific for AML LSCs. For example, treatment with a CD123 antibody impairs cytokine signaling and is toxic to common myeloid precursors (CMPs) [9], and targeting CD44 with an antibody disrupts blast-niche interactions [6].

To identify a more specific marker of AML LSCs, three LSC RNA sequencing datasets and an AML MRD microarray dataset were analyzed. CD9, the most intensively upregulated membrane molecule, was selected as a candidate marker for AML LSCs and has been reported to be involved in several types of CSCs, including pancreatic cancer stem cells, breast cancer stem cells, ovarian cancer stem cells, glioblastoma stem cells, and LSCs in B-acute lymphoblastic leukemia [15, 20, 22, 45, 46].

As a member of the tetraspanin superfamily, CD9 was first identified by Kersey et al. as the human hematopoietic progenitor cell surface antigen p24 using a monoclonal antibody that bound acute lymphoblastic leukemia cells [47]. CD9 has been reported to be expressed in 40% of human AML samples and associated with clinical outcomes in AML [21]. In this study, we demonstrate that CD9 is highly expressed in CD34^+^CD38^−^ AML LSCs and shows extremely low or no expression in normal HSCs, which indicates CD9 to be as a promising therapeutic target in AML. Nevertheless, the evaluation of therapies targeting CD9 still requires further study.

Understanding the underlying mechanisms of CSC maintenance also provides potential for improved patient care and prognosis. For example, Hedgehog (Hh), Notch, and Wnt signaling exhibit significant crosstalk during embryogenesis. Inhibitors of the Hh and Notch pathways have achieved considerable progress in early phase clinical trials [48]. To identify the mechanisms that regulate the characteristics of CD9^+^ LSCs, we performed RNA sequencing of CD9^+^ and CD9^−^ cells from three AML patients. We verified that A2M was involved in regulating the stemness characteristics of CD9^+^ cells. Importantly, it has been reported that activated A2M signals promote the proliferation and survival of cancer cells predominantly through cell surface GRP78 (CS-GRP78) [49]. Therefore, we believe that A2M signaling plays a crucial role in AML and that treatments targeting the A2M signaling pathway will bring new hope to AML patients.

## Conclusion

Our study demonstrated that CD9 is highly expressed in AML LSCs but shows almost no expression in normal HSCs, which allowed it to serve as a potential LSC marker. CD9-positive cells possess CSC characteristics, including drug resistance and migration ability, and promote leukemia progression. Importantly, we found that A2M signaling plays a crucial role in the stemness maintenance of CD9-positive cells in AML. Overall, our results identified CD9 as a new target for AML therapy.

## Supplementary Information

**Additional file 1: Table S1.** Patient characteristics in this study. **Table S2.** Primes for Chromatin Immunoprecipitation in this study. **Table S3.** The 23 genes were significantly upregulated in the MRD microarray.

**Additional file 2: Figure S1.** Flow cytometry was used to detect the expression of CD9 in AML cell lines. **Figure S2.** Flow cytometry detects the cell cycle of CD9^+^ and CD9^-^ cells. *p<0.05, **p<0.01, ***p<0.001. **Figure S3.** Survival curve of mice transplanted with CD9^+^ THP-1 cell and CD9^-^ THP-1 cells (*n*=7) (1X10^6^ cells/mouse). **Figure S4.** ChIP was used to verify the binding of transcription factor EGR1 to the promoter of CD9. *p<0.05, **p<0.01, ***p<0.001.

## Data Availability

For data requests, please contact the authors.

## Abbreviations

LSCs: Leukemia stem cells
AML: Acute myeloid leukemia
A2M: Alpha-2-macroglobulin
HSCs: Hematopoietic stem cells
CD9^−^: CD9-negative
CD9^+^: CD9-positive
MRD: Minimal residual disease
CSCs: Cancer stem cells
CMPs: Common myeloid precursors
FC: Flow cytometry
Ara-C: Cytarabine
