# Supplementary material for: CD9, a potential leukemia stem cell marker, regulates drug resistance and leukemia development in acute myeloid leukemia
Source: Stem Cell Res Ther. 2021 Jan 25;12:86. doi: 10.1186/s13287-021-02155-6 (PMC7836575; doi:10.1186/s13287-021-02155-6)
Supplement: Supplementary file 2 — Additional file 2: Figure S1. Flow cytometry was used to detect the expression of CD9 in AML cell lines. Figure S2. Flow cytometry detects the cell cycle of CD9+ and CD9- cells. *p<0.05, **p<0.01, ***p<0.001. Figure S3. Survival curve of mice transplanted with CD9+ THP-1 cell and CD9- THP-1 cells (n=7) (1X106 cells/mouse). Figure S4. ChIP was used to verify the binding of transcription factor EGR1 to the promoter of CD9. *p<0.05, **p<0.01, ***p<0.001. [file 13287_2021_2155_MOESM2_ESM.docx]

**Supplementary Figures**


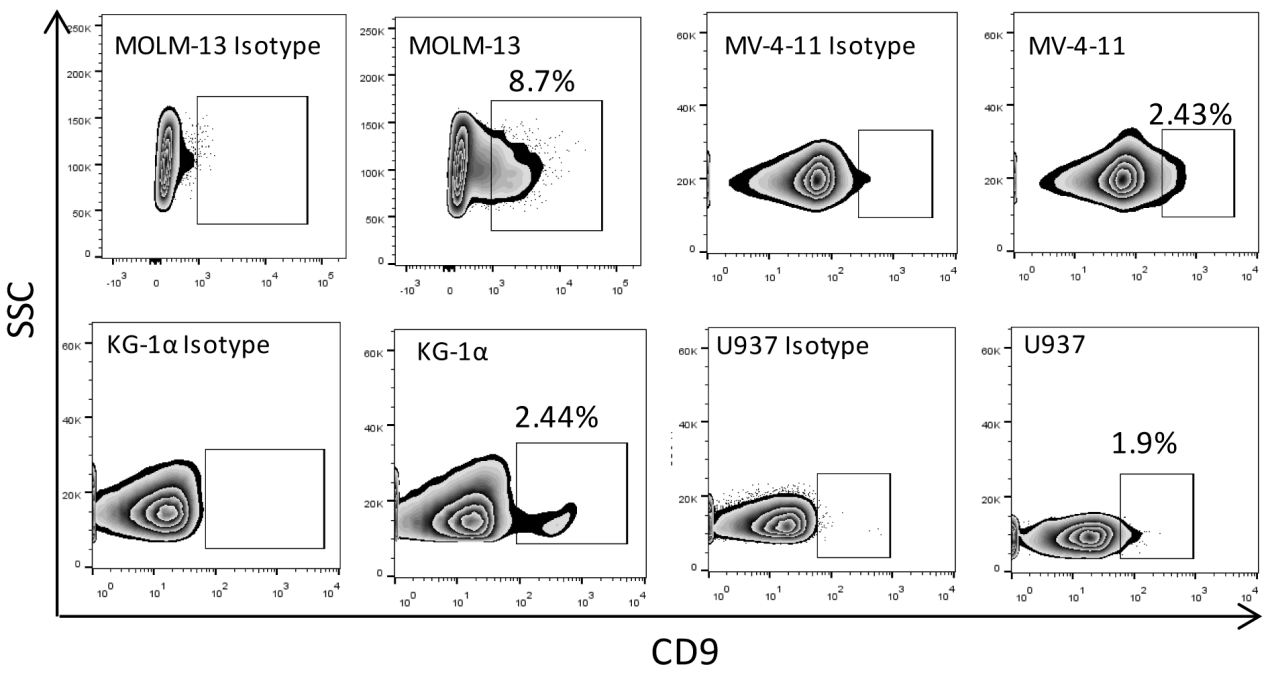


**Figure S1: Flow cytometry was used to detect the expression of CD9 in AML cell lines.**

**
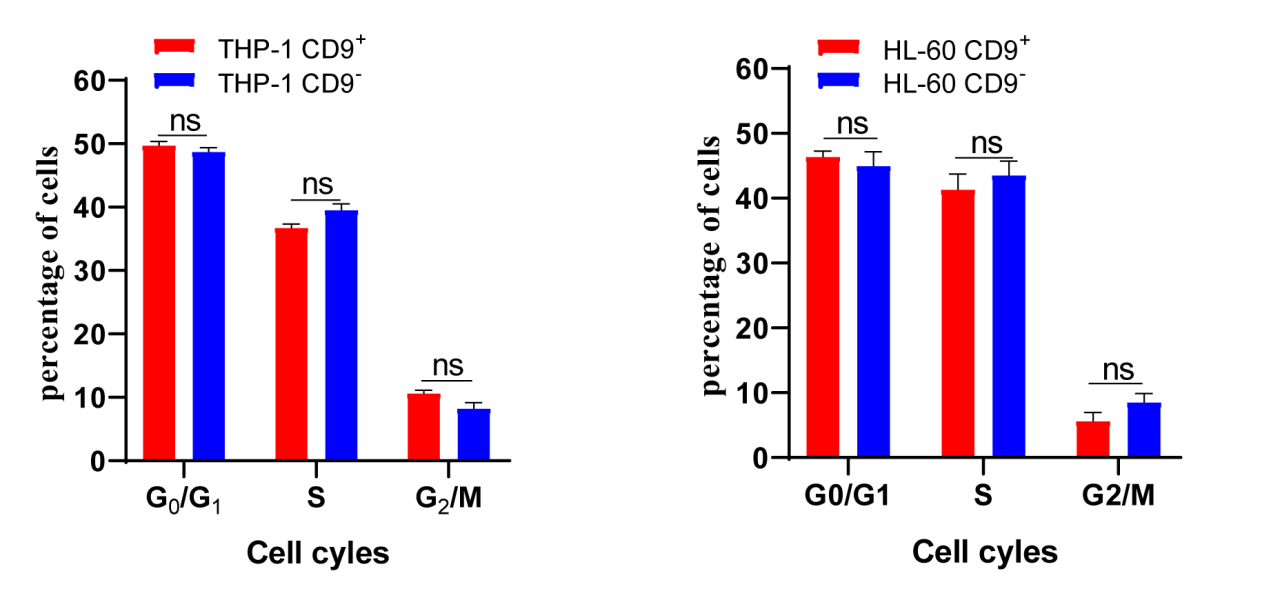
**

**Figure S2: Flow cytometry detects the cell cycle of CD9^+^ and CD9^-^ cells. *p<0.05, **p<0.01, ***p<0.001.**

**
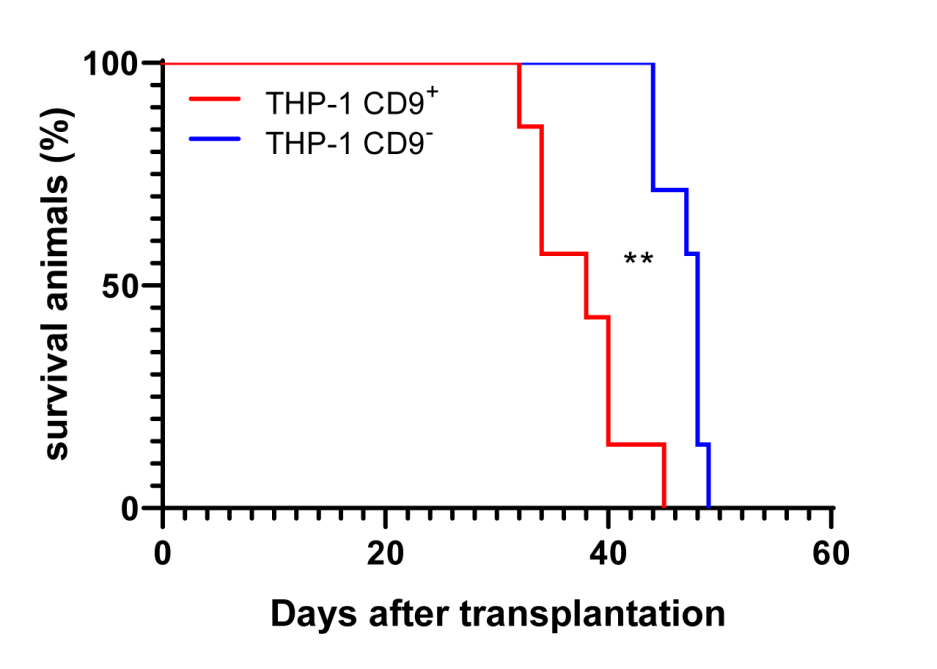
**

**Figure S3: Survival curve of mice transplanted with CD9^+^ THP-1 cell and CD9^-^ THP-1 cells (n=7) (1X10^6^ cells/mouse).**

**
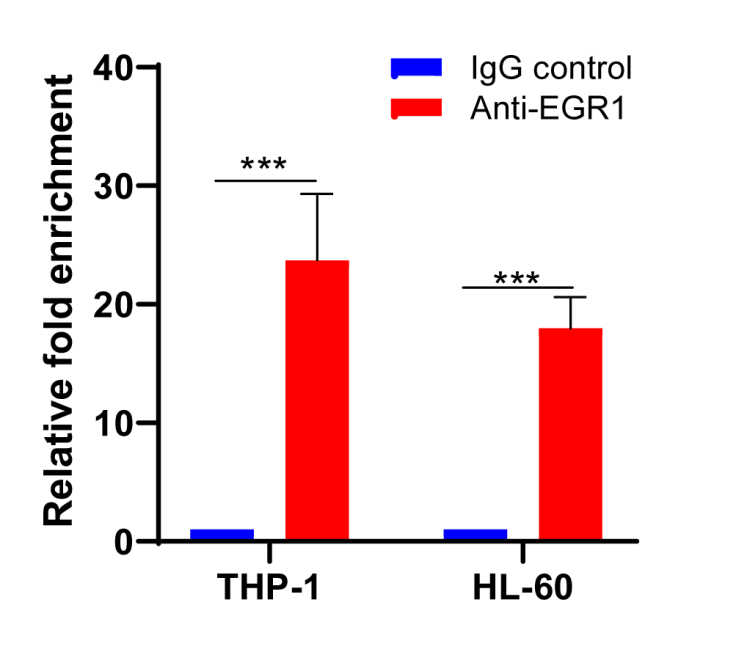
**

**Figure S4: ChIP was used to verify the binding of transcription factor EGR1 to the promoter of CD9. *p<0.05, **p<0.01, ***p<0.001.**
